# Electrocautery-assisted Pacemaker Lead Extraction: A Comparative Evaluation of Cutting Versus Coagulation Modes on a Porcine Muscle Model with Clinical Translation

**DOI:** 10.19102/icrm.2026.17086

**Published:** 2026-08-15

**Authors:** Malugari Anish Reddy, Daljeet Kaur Saggu, Muthiah Subramanian, Sachin Yalagudri, Dinakar Bootla, Sridevi Chennapragada, Prabhakar Reddy, C. Narasimhan

**Affiliations:** 1 Department of Cardiac Sciences, AIG Hospitals, Hyderabad, India; 2 Department of Cardiology, All India Institute of Medical Sciences, Hyderabad, India

**Keywords:** Electrosurgical cautery, lead excoriation, lead extractions, pacemaker infection, pacemaker malfunction

## Abstract

The increasing use of cardiac implantable electronic devices has led to a rise in transvenous lead extractions (TLEs). The most common indications for TLE are infections and lead malfunction. Over time, leads develop fibrotic encapsulation that makes extraction difficult and increases the risk of myocardial injury. Given the thin myocardial wall, particularly in the right atrial appendage and right ventricular apex, the use of advanced powered tools near the lead tip is contraindicated, posing additional challenges. Electrosurgical energy (EE) has been explored as an adjunct to manual extraction to facilitate safe lead tip removal. This study compares the efficacy and safety of cut and coagulation modes of EE using a porcine tissue model and reports its clinical application in a human case. In the porcine model, the cut mode achieved rapid separation of the lead tip but resulted in deeper tissue penetration. In contrast, the coagulation mode (fulguration setting) enabled controlled dissection with minimal depth of penetration without any collateral injury. The application of the coagulation mode during an actual lead extraction with a dwell time of 8 years in a human patient resulted in complete removal with no postoperative complications. These findings suggest that the coagulation mode of EE offers a safer and more controlled technique for lead tip extraction.

## Introduction

The use of cardiac implantable electronic devices (CIEDs) has steadily increased over the last three decades. Despite their therapeutic benefits, pacemaker and defibrillator leads are prone to long-term complications. The most common indication for lead extraction is infection. Other complications such as pocket erosion and lead dysfunction often necessitate extraction.^1^ Lead extraction remains technically challenging due to the development of fibrotic adhesions at key anatomical sites such as the axillary or subclavian vein entry point, innominate vein, superior vena cava (SVC)–right atrial (RA) junction, and the lead tip. Over the past two decades, specific tools and techniques have been developed to facilitate transvenous lead extraction. These have been useful in separating the lead body from the surrounding adhesions.^2^

The Achilles heel of lead extraction is lead tip separation. Even though tools such as TightRail™ (TR) sheath (Philips, Eindhoven, the Netherlands) and excimer laser can be used throughout the entire course of the lead, their utility at the lead tip is limited due to the increased risk of myocardial perforation. Instead, the proposed method of lead tip extraction is the use of counterpressure, traction, and countertraction.^3,4^ However, the major factor limiting extraction success is the thickness of the RA appendage (0.5–2 mm) and right ventricle (RV; 2–5 mm). Fibrosis is typically more extensive around tined lead tips compared with screw-in designs. Increasing lead dwell time further escalates procedural complexity. Specialized tools including locking stylets, telescoping sheaths (eg, metal composition, excimer laser, and radiofrequency [RF] current), snares, and grasping devices are used to remove lead fragments.^2^ The majority of cardiac injuries are due to the overuse of traction.^5^ The use of electrosurgical energy (EE) application at the lead tip can serve as an adjunct to this, thereby decreasing the incidence of myocardial perforation. These risks underscore the importance of employing cautious techniques and meticulous procedural planning to minimize potentially life-threatening complications. EE has emerged as a potential adjunct to traction, with a prior animal study demonstrating improved outcomes when compared with manual traction alone.^6^ Building upon this foundation, we tried to provide a macroscopic overview of the efficacy and safety of cut mode versus coagulation mode of EE in a porcine muscle model, followed by the application of this approach in a clinical case of chronic lead extraction.

## Methods

**Figure 1:**
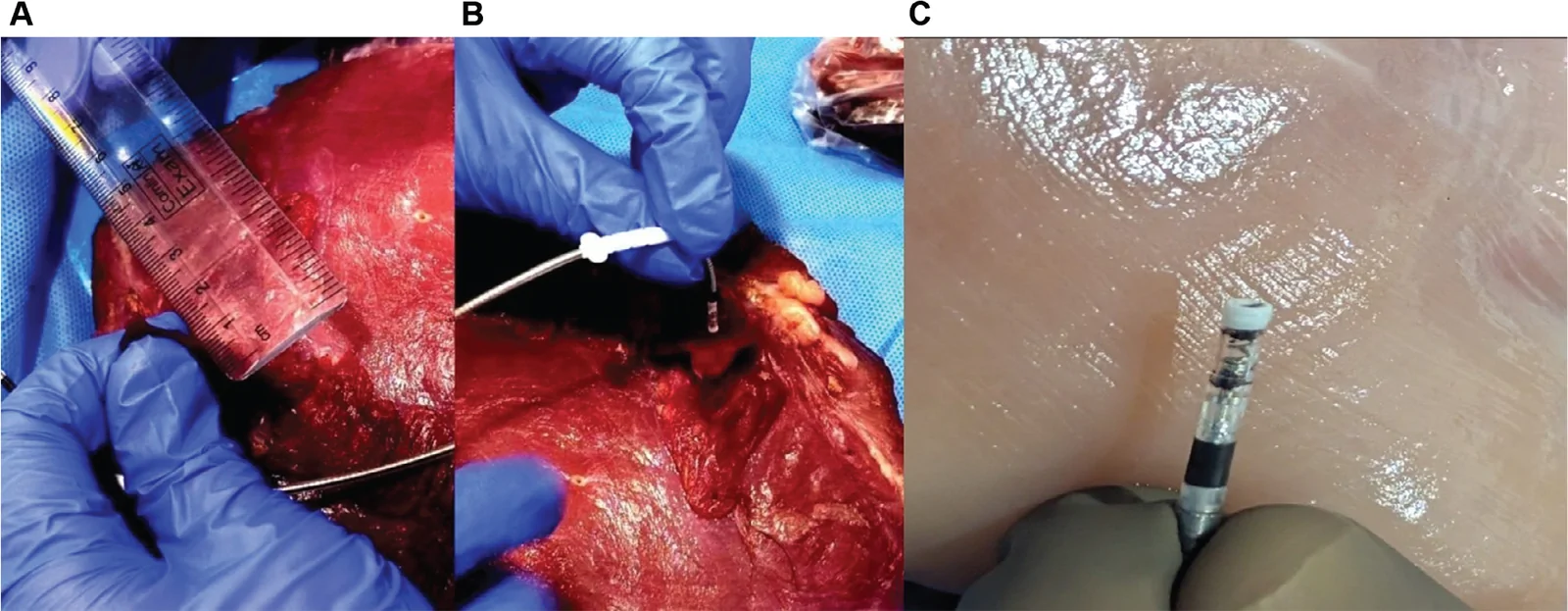
**A:** The cross-sectional thickness of the porcine thigh muscle. It measures up to 2 mm. **B:** The screw-in lead deployed into the muscle tissue. **C:** The lead tip of the screw-in lead with the screw retracted within the sheath.

A porcine thigh muscle model **(Figure 1A)** was used to demonstrate the macroscopic response when cut and coagulation modes were applied in a unipolar cautery to the proximal pin of both tined and screw-in leads. Although we screwed in the screw-in lead to check for the response **(Figure 1B)**, we placed the tined lead on the muscle mass to assess the response to EE. For the screw-in lead, the response was assessed with the screw extended into the myocardium and in the retracted position as well **(Figure 1C)**.

Separately, during a single case, removal of a chronic RV dual-coil screw-in defibrillator lead demonstrating dense fibrotic encapsulation that could not be extracted by conventional means even after the screw was retracted was explored using unipolar electrocautery system in coagulation mode.

The objective of this study was to understand the effect of cut versus coagulation modes of EE on the tips of screw-in and tined leads. Written informed consent was obtained from the patient involved. Ethics committee approval for the article is not applicable.

### Coagulation mode

The coagulation mode typically has three settings **(Table 1)**. It can be switched depending upon the extent of tissue penetration required by the surgeon. As the fulguration setting does not require contact with the tissue, has minimal penetration, and is effective in separating the surrounding fibrotic tissue without any collateral damage, we used the fulguration setting in the coagulation mode.

**Table 1:** Comparison of Coagulation Modes

Method	Electrode Contact	Voltage Level	Nature of Tissue Effect	Clinical Use
Desiccate	Direct contact^18^	Low	Results in the loss of intracellular water and hence dehydration^19^	Precise vessel sealing, controlled bleeding
Fulgurate	Close but not touching^19^	High	Causes carbonization of the tissue, preventing deeper thermal spread^19^	Effective in controlling small bleeding sites^20^
Spray	Further away (larger arc)	High	Superficial widespread coagulation	Broad surface hemostasis

### Settings used in the cut and coagulation modes

For both cut and coagulation modes, the following power settings were used: 25 W, 2 s each, and not more than four cycles each using a Force FX electrosurgical generator (Valleylab, Boulder, CO, USA). During coagulation, the setting was the fulguration mode, as this is associated with least depth and least collateral damage. The grounding electrode was placed beneath the porcine muscle specimen.

## Results

### Experimental findings

**Figure 2:**
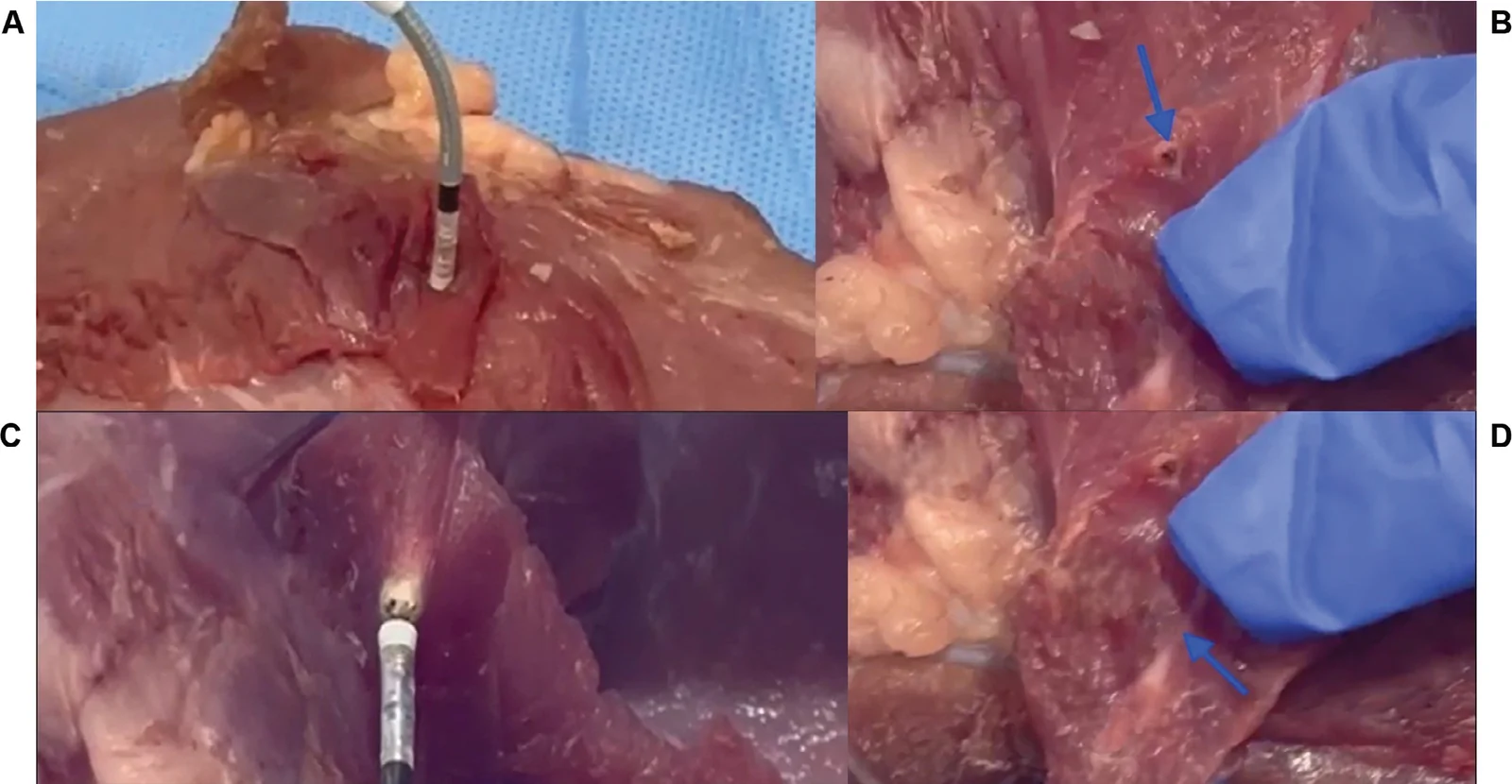
Results with the screw-in lead. **A:** Monopolar cautery applied in the cut mode on the porcine tissue model. **B:** After applying cautery in the cut mode, the lead separated but there was evidence of collateral damage seen at the opposite end. **C:** Monopolar cautery applied in the coagulation mode on the porcine tissue model. **D:** After applying cautery in the coagulation mode, the screw-in lead separated and there was no collateral damage extending to the other end.

**Figure 3:**
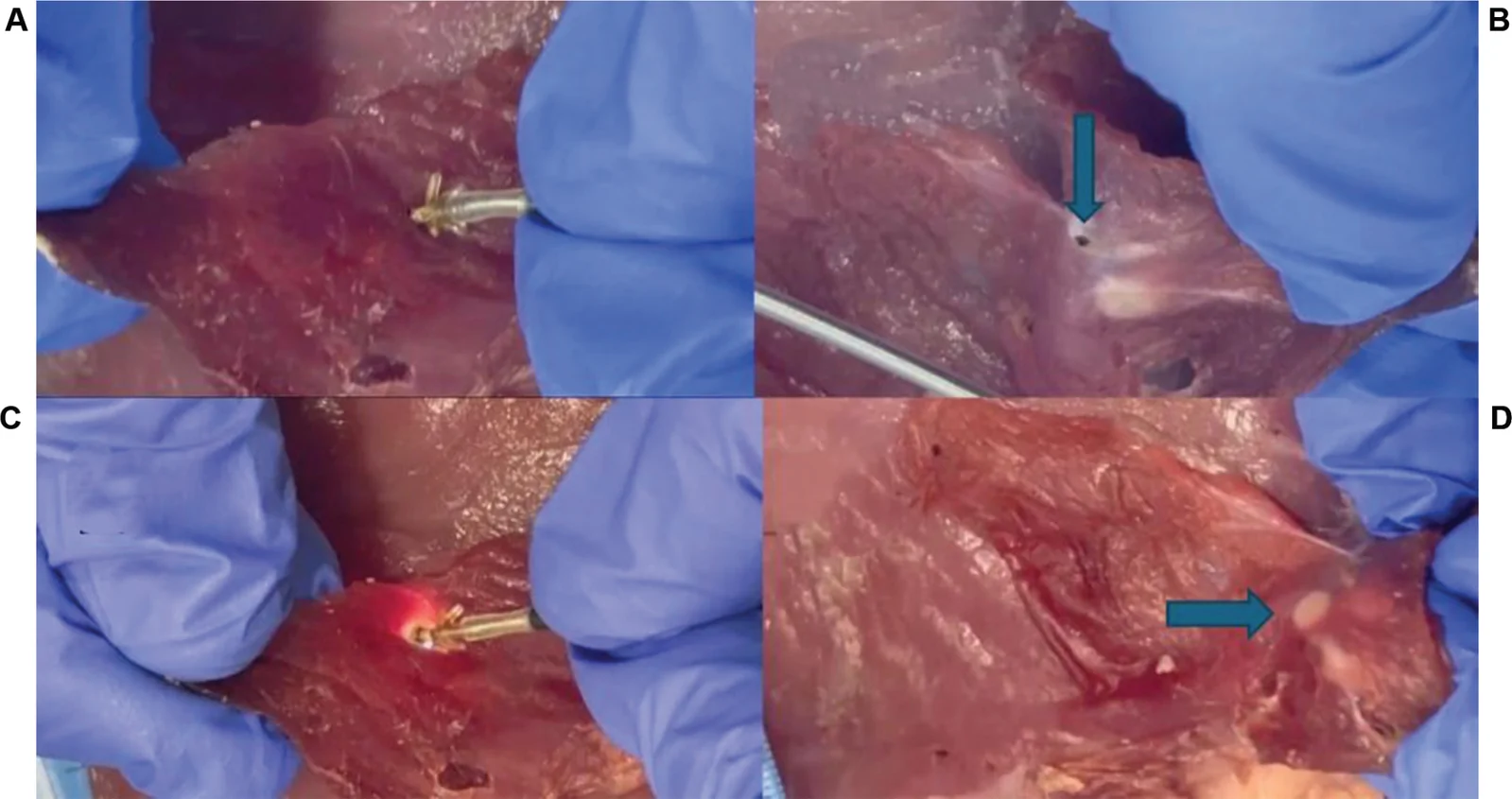
Results with the tined lead. **A:** Monopolar cautery applied in the cut mode on the porcine tissue model. **B:** After applying cautery in the cut mode, collateral damage extending to the other end is seen. **C:** Monopolar cautery applied in the coagulation mode on the porcine tissue model. **D:** After applying cautery in the coagulation mode, there was no collateral damage extending to the other end.

**Figure 4:**
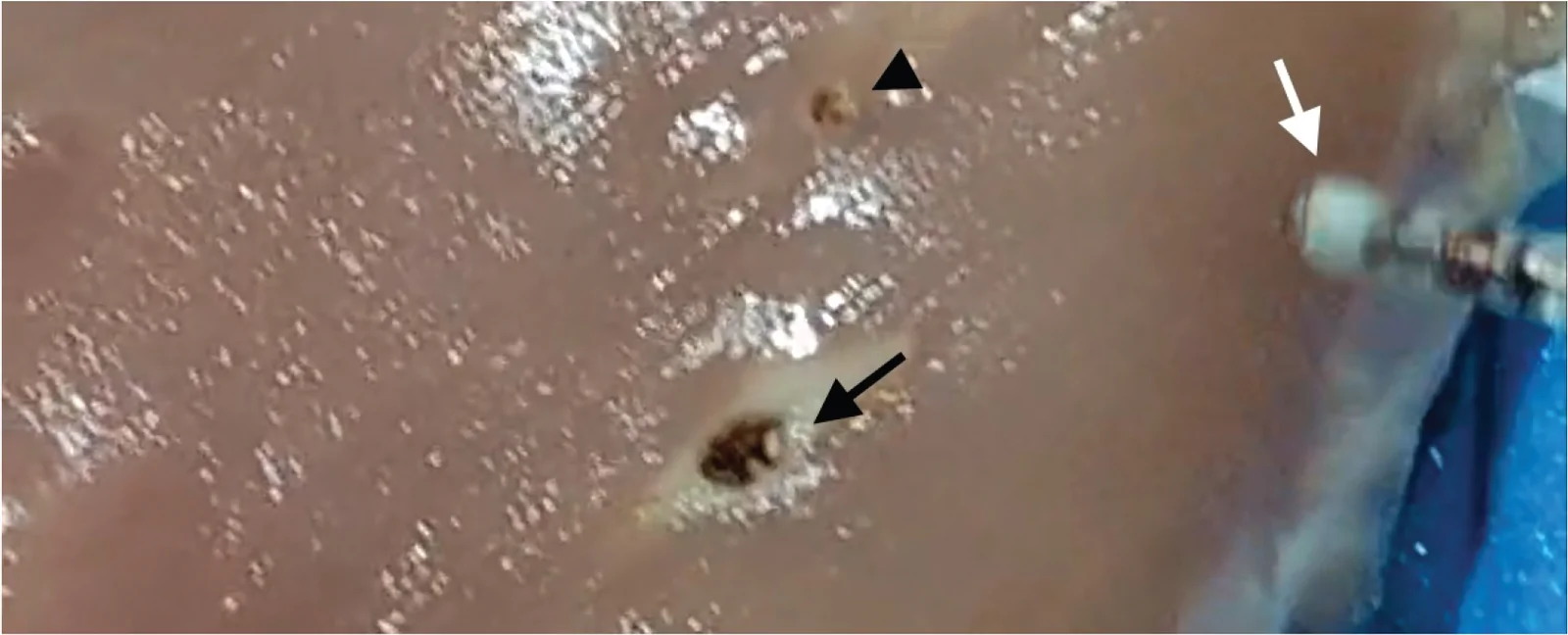
The effect of monopolar cautery in the coagulation mode applied to the screw-in lead (white arrow) with the screw both in deployed (black arrow) and retracted positions (black arrowhead). We can appreciate the extent of tissue separation created with the screw in the deployed position (black arrow) versus screw in the retracted position (black arrowhead). Despite the screw being in the retracted position, it does create an effect on surrounding tissue. See text for details.

In the cut mode, the screw-in lead was released after two cycles of EE; however, this was accompanied by perforation of the surrounding tissue, indicating uncontrolled energy delivery and collateral injury **(Figures 2A and 2B)**. When applied to the tined lead, the cut mode again resulted in tissue perforation along with a tunneling or drilling effect of the lead, as demonstrated in the experimental recordings **(Figures 3A and 3B and Video 1)**. In contrast, application of EE to the tined lead tip in the coagulation mode facilitated controlled separation of the surrounding tissue without evidence of perforation or tunneling effects **(Figures 3C and 3D and Video 2)**. The screw-in lead tip was successfully released after three cycles of coagulation mode, with no tunneling or drilling effect. **Figure 4 and Video 3** demonstrate the transfer of EE energy to the lead tip of the screw-in lead in its retracted form. Despite the retracted screw being covered by a protective sleeve, it separated surrounding tissue fibers both in cut and coagulation modes (although to a lesser extent). Thus, from this experiment, we can infer that EE can be considered as an adjunctive tool during lead extraction, even if the screw is in the retracted form and the lead is stuck because of chronic fibrosis.

These findings suggest that EE in the coagulation mode (fulguration setting) enables safe and controlled separation of the lead tip from the surrounding tissue, thereby reducing the risk of myocardial injury. Based on these experimental results, we subsequently applied this technique in a clinical lead extraction case, which is described in detail below.

### Clinical application

A 55-year-old man with ischemic cardiomyopathy and a history of biventricular pacing presented with infective endocarditis and device pocket infection. He had originally received a dual-coil screw-in defibrillator lead in June 2017, which was subsequently upgraded to a biventricular defibrillator in March 2025. Three months after the device upgrade, he developed a persistent fever and a local pocket infection. Despite an initial course of antibiotics, there was no significant clinical improvement, necessitating complete system extraction. The recently implanted RA and left ventricular screw-in leads (dwell time of 3 months) were successfully removed by manual traction. However, the chronic RV dual-coil screw-in defibrillator lead (dwell time of 8 years) demonstrated dense fibrotic encapsulation and could not be extracted by conventional means even after the screw was retracted. Guided by our preclinical findings, we used a unipolar electrocautery system in coagulation mode applied to the proximal lead pin (power, 25 W; fulguration setting; cycle duration, 2 s; two applications). Following this, we shredded the proximal 3–5 cm of insulation with a no. 11 blade, leaving the lead pin intact. Also, with the support of locking stylet no. 2 and 13-Fr TR, adhesions surrounding the subclavian vein, innominate vein, and SVC–RA junction could be removed. Once the TR separated the SVC coil from surrounding adhesions, the lead tip came out easily, without any further traction. This approach facilitated complete lead removal without evidence of myocardial or vascular injury. The patient tolerated the procedure well, and no intra- or post-procedural complications were observed. Therefore, we suggest that, if traction alone proved insufficient, a unipolar electrocautery system in coagulation mode can be applied to the proximal lead pin to loosen the surrounding adhesions. Additionally, in our porcine experiment model, we realized the technical limitation of using EE after loading the lead with a lead-locking device (LLD) and TR. This was because of the length discrepancy between the TR (68 cm) and average RA/RV leads (52–60 cm). Hence, during lead extraction, we initially attempted manual extraction and then used EE before the application of TR and LLD.

## Discussion

EE is extensively used in major surgical procedures. For CIED implantation, it is predominantly used for pocket creation. The cut mode in EE is used when tissue division is intended, and the coagulation mode is deployed when hemostasis is desired. In this study, we extended its use as an adjunct tool for lead tip extraction, comparing cut and coagulation modes. In the porcine muscle model, the cut mode (two cycles) effectively separated the lead tip from surrounding tissue but was associated with collateral injury extending to the other side of tissue **(Video 1 and Figures 2, 3A, and 3B)**. In contrast, the coagulation mode (three cycles) applied using the fulguration setting demonstrated minimal tissue penetration and the absence of any collateral injury, indicating a more favorable safety profile **(Video 2 and Figures 2, 3C, and 3D)**. We also performed this experiment on the tip of the screw-in lead with the screw in the retracted position. Despite the screw being covered by a protective sleeve, we could demonstrate the effects of EE in both cut and coagulation modes **(Video 3 and Figure 4)**. Guided by these experimental findings, EE in coagulation mode with the fulguration setting was successfully applied during the extraction of a chronically implanted screw-in RV defibrillator lead with dense fibrotic encapsulation.

Catanzaro et al.^6^ performed an elegant experiment on the feasibility of site-specific EE delivery in lead extraction. They used a polyacrylamide gel model to simulate soft tissue density and repeated the experiment in an acute pig model. They worked mainly on the amount of force required to extract these leads before and after application of EE. However, they did not study the mode or duration of cautery settings to understand the safety profile. In contrast, we used a thin porcine muscle sheet **(Figure 1A)** to demonstrate the effect of different modes of EE along with their collateral effects and translated it into clinical use. Talreja et al.^7^ demonstrated the successful use of RF ablation applied at the tip of a chronically implanted tined lead that had failed to be extracted with other standard methods. Luo et al.^8^ studied the different modes and settings of RF energy applied to the SelectSecure 3830 lead (Medtronic, Minneapolis, MN, USA) in excised swine heart models. Under fluoroscopic guidance, the lead was positioned against the interventricular septum. They used this technique to understand the safe RF energy settings, which can be used to penetrate the lead through the fibrosed septum to reach the left bundle branch area without causing perforation. Studies have reported that the use of mechanical rotational sheaths has resulted in major complication rates between 0% and 1.5% with lead dwell times around 7.1–9.1 years,^9–12^ while laser extraction had reported complication rates of 0.9%–2.5% with dwell times of around 5.4–6.8 years.^13–17^ The integration of EE into the extraction process may help mitigate these risks, particularly in leads with prolonged dwell times or dense fibrosis. With the concurrent use of EE at the lead tip, the amount of traction and countertraction required to separate the lead tip safely from the fibrosed myocardium may decrease. The easy availability of EE in our operation theaters/catheterization laboratories without added cost to patients makes it a viable option.

In summary, our findings demonstrate that EE in coagulation mode, specifically with fulguration settings, represents a promising adjunct to conventional lead tip–extraction techniques. This novel approach may expand the armamentarium of tools available for managing chronically implanted leads where conventional techniques are limited by the risk of myocardial injury.

### Limitations

The porcine skeletal muscle model does not fully replicate the intravascular and intracardiac environment where lead fibrosis typically develops. In clinical practice, adhesions occur within vascular and myocardial structures, and a simplified experimental setting may not replicate the cardiac environment. We acknowledge that this is an acute single time-point study on fresh skeletal muscle tissue without any fibrosis around. However, in this study, we demonstrated the tissue response to different modes of electrocautery applied to both the tined and screw-in lead tips (in retracted and extended positions) to understand its safety profile. Moreover, the experimental protocol did not entirely reflect standard clinical conditions. In the porcine muscle tissue experiment, we tried to demonstrate the effects of cautery by applying a pushing force on the tined and screw-in leads in their retracted form, in contrast to pulling the leads away from the myocardium. The tined lead was placed on, rather than implanted into, the muscle mass, thereby limiting the assessment of EE effects on true fibrotic encapsulation. In addition, tissue response was evaluated only macroscopically without histopathological analysis, precluding precise characterization of thermal spread or collateral injury. Finally, although the clinical case demonstrated successful and uncomplicated extraction, a single case may not establish reproducibility and safety. Larger clinical studies are necessary to validate this approach. Despite all the limitations, however, we believe that this study has paved the way for future studies with better experimental models.

## Supporting information

Supplementary Video 1: Shows the application of cautery applied to the tined lead tip in cut mode on porcine muscle tissue. It shows that when cautery is used in cut mode it pierces through the muscle tissue.

Supplementary Video 2: Shows application of cautery applied to the tined lead tip in coagulation mode on the porcine muscle tissue. It shows when cautery is used in coagulation mode it does not cause a perforation and is limited to only separation of surrounding tissue from the lead.

Supplementary Video 3: Shows the effect of EE in cut mode applied to the screw-in lead tip with the screw retracted within the sheath. Despite of screw covered with the sheath it does show the effect of EE on the muscle tissue.
